# The CONSTANCES cohort: an open epidemiological laboratory

**DOI:** 10.1186/1471-2458-10-479

**Published:** 2010-08-12

**Authors:** Marie Zins, Sébastien Bonenfant, Matthieu Carton, Mireille Coeuret-Pellicer, Alice Guéguen, Julie Gourmelen, Mélissa Nachtigal, Anna Ozguler, Ariane Quesnot, Céline Ribet, Grégory Rodrigues, Angel Serrano, Rémi Sitta, Alain Brigand, Joseph Henny, Marcel Goldberg

**Affiliations:** 1 Inserm U1018, Epidemiology of occupational and social determinants of health - Centre for Research in Epidemiology and Population Health, 16 avenue Paul Vaillant Couturier, F-94807, Villejuif, France; 2 Versailles-Saint Quentin University, Versailles, France; 3 Centre d'examens de santé de Saint Brieuc-CPAM des Côtes d'Armor, Côtes d'Armor, France; 4 Centre de médecine préventive de Vandœuvre les Nancy, Vandœuvre les Nancy, France

## Abstract

**Background:**

Prospective cohorts represent an essential design for epidemiological studies and allow for the study of the combined effects of lifestyle, environment, genetic predisposition, and other risk factors on a large variety of disease endpoints. The CONSTANCES cohort is intended to provide public health information and to serve as an "open epidemiologic laboratory" accessible to the epidemiologic research community. Although designed as a "general-purpose" cohort with very broad coverage, it will particularly focus on occupational and social determinants of health, and on aging.

**Methods/Design:**

The CONSTANCES cohort is designed as a randomly selected representative sample of French adults aged 18-69 years at inception; 200,000 subjects will be included over a five-year period. At inclusion, the selected subjects will be invited to fill a questionnaire and to attend a Health Screening Center (HSC) for a comprehensive health examination: weight, height, blood pressure, electrocardiogram, vision, auditory, spirometry, and biological parameters; for those aged 45 years and older, a specific work-up of functional, physical, and cognitive capacities will be performed. A biobank will be set up. The follow-up includes a yearly self-administered questionnaire, and a periodic visit to an HSC. Social and work-related events and health data will be collected from the French national retirement, health and death databases. The data that will be collected include social and demographic characteristics, socioeconomic status, life events, behaviors, and occupational factors. The health data will cover a wide spectrum: self-reported health scales, reported prevalent and incident diseases, long-term chronic diseases and hospitalizations, sick-leaves, handicaps, limitations, disabilities and injuries, healthcare utilization and services provided, and causes of death.

To take into account non-participation at inclusion and attrition throughout the longitudinal follow-up, a cohort of non-participants will be set up and followed through the same national databases as participants.

A field-pilot was performed in 2010 in seven HSCs, which included about 3,500 subjects; it showed a satisfactory structure of the sample and a good validity of the collected data.

**Discussion:**

The constitution of the full eligible sample is planned during the last trimester of 2010, and the cohort will be launched at the beginning of 2011.

## Background

Large-scale, prospective observational cohort studies that include several hundreds of thousands of individuals and that incorporate personal, social, lifestyle, occupational and environmental data as well as biobanks of blood and other biological specimens have become essential resources for studies on the causes of and major risk factors for many diseases. Since the Framingham Study followed-up from 1948 on [1], many larger prospective cohorts were launched in different countries, such as the Nurses' Health Study [2], the One Million Women Study [3], the UK Biobank [4], the Kadoorie Study in China [5], or the EPIC European Prospective Investigation into Cancer and Nutrition [6].

### Main objectives

The objective of the CONSTANCES ("Cohorte des consultants des Centres d'examens de santé") project is to set up a large population-based cohort to contribute to the development of epidemiologic research and to provide useful public health information. It is conducted in partnership with the National Health Insurance Fund administered by the *Caisse nationale d'assurance maladie des travailleurs salaries *(CNAMTS) [7], the principal health insurance fund in France, which covers more than 80% of the French population, and with the Ministry of Health and the National Institute of Health and Medical Research. This cohort is intended to serve as an "open epidemiologic laboratory" widely accessible to the epidemiologic research scientific community; due to the open access provided to the community of researchers, it will be possible to conduct projects on a variety of scientific questions. The CONSTANCES cohort will be a large sample, representative of the general French population, and characterized by broad coverage of health problems and health determinants. It will serve as an important scientific instrument, in a similar manner to a telescope or a particle accelerator, for example, or a genotyping laboratory with sequencers ― built not to answer a specific question but rather to help analyze a wide range of scientific problems. In this regard, the design of CONSTANCES relied on the experience of the GAZEL Cohort Study, an open general-purpose prospective cohort established in 1989 by our research unit which is currently supporting more than 40 different nested research projects on very diverse scientific topics [8-10].

CONSTANCES was also designed as a tool to support the public health objectives of the national government. Indeed, numerous data sources in France provide public health officials with some of the information they need. Nonetheless, these all have limitations. One often-highlighted gap is the lack of large longitudinal studies covering a broad range of health outcomes, taking into account the changes over time of people and the socioeconomic environment [11,12]. CONSTANCES, by its thorough system for the follow-up and collection of very diverse information through a variety of methods and data sources on a large representative sample of the adult population, will contribute to a better knowledge of the health of the French population.

### Specific research themes

Although designed as a general-purpose cohort intended to host numerous nested projects with a very broad scope, the major orientation of CONSTANCES is the study of occupational and social determinants of health. These themes are essential areas of research in public health and epidemiology today, involving numerous health problems and diverse populations.

Health risk factors with origins in occupational exposures are numerous: chemical hazards, noise, temperature, vibrations, radiation, biological agents, physical and postural constraints, mental load and stress, hours, and work pace. These exposures affect a high percentage of the population. Psychosocial factors linked to the organization of work and to an imbalance between the individual's efforts and rewards are also sources of potentially pathogenic stress [13,14]. Numerous studies have shown the role of psychosocial factors at work in cardiovascular disease, in the incidence of mental disorders, including depression, on quality of life, and in musculoskeletal diseases [15]. The way that companies are run, including, for example, the increasingly frequent use of subcontractors, fixed-term contracts, and temporary workers, creates different processes that increase job insecurity and affect living conditions. Little is known about the relations between these processes and health. Working conditions, occupational exposures, and life-long occupational trajectory are also major determinants of the aging process. The disorders that over time lead to impairments, disabilities, and diseases most often originate early in working life and build up over time until they become chronic. The same is true of occupational constraints and hazards. All may lead to an early exit from the labor market. Finally, occupational factors are also major determinants of social inequalities in health.

In France, the system of universal access to health care corresponds to an ideal of equality with regards to disease and death. Nonetheless, while health status improves generally in the population, social inequalities in health persist, and some have even worsened [16]. Moreover, this phenomenon is not limited to the degradation of health status among the most disadvantaged groups. Rather, there is increasing evidence of an inverse gradient between various measures of socioeconomic position and diverse health problems [17]. These observations have renewed questions about the causes of these inequalities [18] and about their public health implications; if policies to combat these health inequalities are limited to groups described as "insecure" or disadvantaged, they will be globally ineffective. From a public health point of view, developing and implementing policies aimed at reducing social inequalities in health requires a better description of the distribution of health problems of populations, particularly the use of diverse criteria for social status. This knowledge will help foster a better understanding of the nature of the determinants of the disparities observed and the pathways by which they work [19]. Epidemiologic analysis of the social and occupational determinants of health is therefore a major issue, from both scientific and public health perspectives.

Epidemiologic data remain sparse on the topic of changes in health with age and more particularly about aging and its relation to health, work, and the life course. Studies are essentially limited to the age groups above 65 years and provide little information about earlier life periods [20], even though factors that lead to impairments, disabilities, and chronic diseases at advanced ages often begin early in life, and they continue to accumulate throughout life. We still know little about social inequalities in the aging process or about the respective roles played by individual susceptibility factors ― especially genetic factors, exposures across the life course, and living and working conditions in all their dimensions, the conditions in which people stop working, their attention to their health, and primary, secondary and tertiary prevention. The aging-related scientific objectives in CONSTANCES involve the study of the role of various types of risk factors throughout the life course. Another objective is to document the determinants of health most frequently encountered or suspected in chronic age-related diseases. Longitudinal follow-up offers broad possibilities for dynamic study of the delayed effects of living and working conditions on aging (e.g., frailty, cancer, chronic diseases, and mental health). It will also allow study of the factors that may lead to inactivity and isolation, factors and mechanisms that contribute to successful aging, and conversely those that contribute to disabilities and/or frailty [21].

## Methods/Design

### Cohort composition, representativeness and selection effects

The source population is that of the people in France whose health insurance is administered by the CNAMTS. Health insurance is compulsory in France, and all salaried workers and their family are affiliated to this fund, which covers more than 80% of the French population (approximately 50 million people).

As one of the main study objectives is to provide information on the health status and disease burden of this large part of the French adult population, the CONSTANCES cohort will be a representative sample of the general French population insured by CNAMTS in terms of age (18 to 69 years at inception), sex, and social category.

To be able to answer the many questions raised in varied domains, CONSTANCES must be a large sample. In order to assess the potential of CONSTANCES in terms of its capacity to conduct epidemiologic studies likely to have good statistical power, we estimated the number of major health outcomes expected in the CONSTANCES cohort over a moderately long term in a cohort with an age and sex structure identical to that of the French general population aged 18 to 69 years at the 1999 census. Table 1 presents the number of expected events at the end of 5, 10, and 15 years for events for which we have reliable national reference data: deaths and incidence of cancer, ischemic heart disease, and Alzheimer disease. For these major outcomes, the number of these serious events is high and will make possible numerous studies with satisfactory power.

**Table 1:** Expected number of major health outcomes during follow-up of the CONSTANCES cohort*

	5-year follow-up			10-year follow-up			15-year follow-up		
	Men	Women	Total	Men	Women	Total	Men	Women	Total
Death, all causes	4131	2133	6264	9727	5502	15 229	16 983	10 736	27 719
Incident cancers	3162	2220	5381	7036	4855	11 892	11 444	7823	19 267
Ischemic heart disease (35-64 years)	681	138	819	1418	290	1708	2178	452	2630
Alzheimer disease	265	240	505	793	1007	1800	1548	2469	4018

*Reference rates: [33,51,52]

Because the advantages of longitudinal follow-up increase with its duration and in view of the broad objectives for this cohort, the duration of follow-up should be as long as possible; CONSTANCES planned duration is therefore indefinite. A major concern of long-term prospective cohorts is attrition, potentially inducing biases and affecting the power of the study [22]. It is not possible at this stage to estimate precisely the number of subjects who will be lost to follow-up in the CONSTANCES cohort over the years. We can nonetheless makes estimates based on experience with the follow-up of other French cohorts, such as GAZEL, which began in 1989 with more than 20,000 subjects [8-10]. Active participation by self-administered questionnaire is high: after 20 years of follow-up, only 3.1% of the subjects who participated at inclusion never returned an annual questionnaire. The number of subjects truly lost to follow-up, that is, whom we can no longer locate in the databases, is tiny: 107, or approximately 0.5% [9]. It is reasonable to think that CONSTANCES, which will apply similar methods, will also have very high follow-up rates.

One of the major sources of bias in epidemiologic surveys comes from selection effects, which can bias estimates of disease prevalence or incidence (or of prevalence of exposure to a risk factor) and of associations between exposures and diseases of interest. In longitudinal cohorts, selection effects may occur at inclusion and throughout follow-up because of cohort attrition [22]. The problem of bias linked to selection effects is very different depending on whether the objectives are analytic or descriptive [23].

In a cohort whose inclusion procedures are the same for all subjects (the case of CONSTANCES), in principle the exposure-disease relation does not differ between subjects who are included and those who are not [24-26]. Therefore, the selection procedures at inception for CONSTANCES participants should generate minimal bias, if at all, in analytic studies. On the other hand, the problem of attrition during follow-up may cause substantial bias if the probability of continued follow-up is different in exposed and unexposed subjects or in those who do or do not become ill; this is often the case [27].

For descriptive studies of the frequency of health problems and exposures, the parameters of interest must be estimated in a representative sample of the target population. In this regard, the potential concerns for CONSTANCES are mainly incomplete geographical coverage of the districts of recruitment and factors associated with voluntary participation.

**Figure 1:**
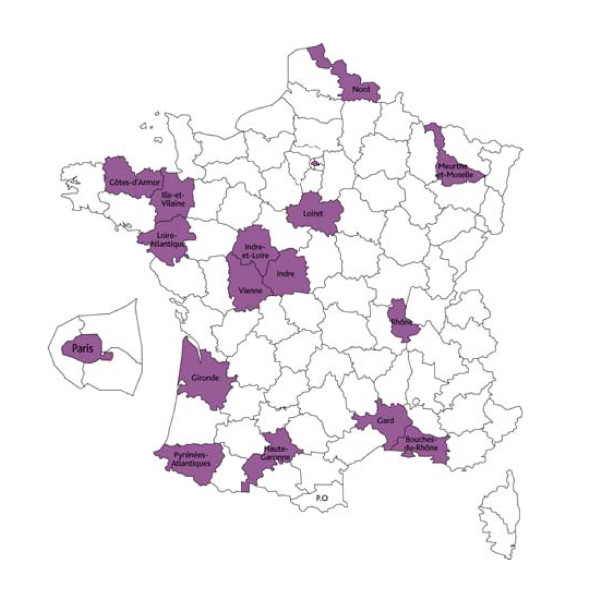
Geographical location of the participating HSCs in France

As detailed below, the cohort participants will be included in 17 Health Screening Centers (HSCs) located in 16 different districts in different regions of France (Figure 1).

We have verified that the structure of the population of the districts where the CONSTANCES HSCs are located is essentially identical to that for France as a whole for the principal demographic, social, and occupational characteristics; we should thus be able to generalize the CONSTANCES results to the French population as a whole (results not shown).

Using volunteer subjects inevitably produces selection effects, even in studies that use random drawing from an appropriate sampling base, as it is the case of CONSTANCES (see below). At inclusion, individuals may refuse participation (become non-participants), a potential source of bias. To compensate, researchers usually attempt to collect a minimum data set for the non-participants (mainly age, sex, and social category), to facilitate subsequent adjustments for estimating the relevant parameters. This approach nonetheless has some limitations. First, it is not always possible to collect the adjustment data for non-participating subjects. Nor is it always clear whether these data are sufficient to control for potential bias, because we know, for example, that within the same socioeconomic category there are many important differences in terms of health, behavior, lifestyles, social networks, etc. [28,29]. Finally, it is rarely possible to control completely for potential selection bias because it is rare to have the relevant data collected simultaneously for the participants and the non-participants.

To obtain a representative sample of the target population and to minimize the bias associated with selection effects at inclusion and during follow-up in CONSTANCES, we will take the following steps:

The sampling base at inclusion is composed of all persons aged 18 to 69 years and covered by CNAMTS in the catchment areas of the 17 CONSTANCES HSCs. Sampling will be done within the database of the National Retirement Insurance Fund administered by the *Caisse nationale d'assurance vieillesse *[30] which includes exhaustively all the persons in France affiliated to the CNAMTS and permanently collects professional and social data compulsory for establishing the retirement benefits of all individuals in France from different sources (see below). The random drawing is stratified according to unequal inclusion probabilities, based on data from participation in previous surveys involving invitations to the HSC [31]. We will also set up a "parallel cohort" from a sample of non-participants for whom we will prospectively collect data on social and demographic characteristics (sex, age, work status, social category), through the CNAV database, as well as information about health and health-care utilization from two other national databases covering the whole French population: the *Système d'information de l'assurance maladie *(SNIIR-AM), the National Health Insurance Information System [32] and the National Death Registry managed by the *Centre d'épidémiologie des causes de décès *(CepiDc) [33]. As we will have data from the SNIIR-AM, CNAV and National Death Registry files for both the participants and the sample of non-participants, we will be able to estimate the probabilities of participation in CONSTANCES with prediction models; the inverse of the probability of participation will then provide an adjustment coefficient for each participant. We can assume that almost none of the people included in CONSTANCES will be permanently lost to follow-up, since the participants will be followed "passively" (so-called because this follow-up will not require the subjects' participation) through the SNIIR-AM, CNAV and National Death Registry files.

There will nonetheless be attrition due to the failure to return the annual questionnaire. Thus, adjustment for attrition is necessary if the analyses from the questionnaires variables are to be valid. Inclusion in CONSTANCES will take place over a 5-year period (see below). For wave 1, we will distinguish the participants who returned the self-administered questionnaire in year 2, that is, the year following their inclusion (which is year 1), from those who did not. We will have the data collected at inclusion in CONSTANCES (year 1) for all participants as well as the SNIIR-AM and CNAV data corresponding to year 1 or to year 0 (the year before inclusion). The coefficients of adjustment for attrition in year 2 can thus be calculated by a method similar to the one used to calculate the coefficient of adjustment for non-participation. For the following years, we will again distinguish the participants who returned the self-administered questionnaire from those who did not. Thus, the longitudinal follow-up of participants in the SNIIR-AM and CNAV databases, whether or not they drop out of the cohort by not returning the annual questionnaire, will make it possible to update the coefficients of adjustment for attrition.

For descriptive purposes, each year we will adjust the cohort data on the reference population. The first year, we will randomly draw from the CNAV files a sample of CNAMTS members in the CONSTANCES districts aged 18 to 69 years. The second year, and respectively the third, fourth and fifth years, this sample of randomly drawn individuals will include people aged 18 to 70 years, then 18 to 71 years, 18 to 72 years and 18 to 73 years. Each of these samples will be twice as large as that of the population of CONSTANCES participants so that the reference population will be significantly greater than the sample. After linkage of this file with the SNIIR-AM files, we will calculate the relevant margins and thus, beyond socioeconomic and demographic characteristics, be able to integrate the variables relative to the health and healthcare utilization characteristics (information also available for the survey participants). The quality of weighting will be therefore substantially improved by the calculation of margins specifically related to health, the focus of the CONSTANCES cohort.

### Procedures for inclusion

Everyone with health insurance from CNAMTS, as well as their dependents, is entitled to receive health examinations that include free extensive work-ups conducted in selected HSCs. Overall the HSCs conduct approximately 600,000 health examinations annually. Randomly selected persons will receive an invitation to come to their HSC for inclusion in the CONSTANCES cohort. The 17 selected HSCs are distributed throughout France; they have experience with the recruitment of large numbers of people and with participating in epidemiological studies. All are large, have a staff motivated to work in epidemiology, and use advanced medical equipment; their geographic distribution represents the principal regions of France (see Figure 1).

We will proceed gradually to the inclusion of the entire cohort over a 5-year period. Each wave will include 40,000 subjects in a year, and the final cohort will be constituted at the end of this 5-year period.

### Procedures for longitudinal follow-up

An annual self-administered questionnaire will be sent to the subjects at home. Post office procedures make it possible to obtain regular updates of participants' postal addresses. Maximizing their personal participation rate is essential. Accordingly, regular contact with participants will include a CONSTANCES Cohort Journal, which will present results, nested projects, etc., and will be sent regularly to participants. A website will also be created.

The subjects included in CONSTANCES will also be followed up passively for social and work-related events and health data by regular linkage with the national databases.

The CNAV databases are essential for access to social and work-related data [30]). This agency's role is to ensure the rights to pension payments after retirement for every individual in France who had health insurance from CNAMTS at least once during his or her life. It has therefore set up a system that allows it to collect social data from different organisms and schemes that manage various forms of insurance and other social protection. The CNAV regularly receives for its databases employers' annual reports, and information about periods of employment and unemployment from social welfare organizations (e.g., sick leave, maternity leave, unemployment, and diverse social benefits).

Access to the SNIIR-AM [32], which covers the entire French population, should be an efficient method of obtaining information about health events. The SNIIR-AM contains individual medical data from different sources, structured and coded in a standardized manner: reimbursement data (doctors and other health professionals visits, prescribed drugs); so-called "long-term diseases" (serious diseases exempt from co-payments and user fees, coded according to the International Classification of Diseases 10th revision - ICD 10 [34]); hospital discharge records, including principal and associated diagnoses for each hospitalization, also coded according to ICD 10. Vital status and causes of death will be obtained from the National Death Registry.

### Principal data to be collected from different sources

Here we summarize the main data to be collected from different sources (self-questionnaires, medical examination, national health and social databases), at each stage of the study; the detailed list of data can be downloaded from CONSTANCES' website [35]. When possible, we selected variables already used in other surveys, both because they are validated measures and because it will thus be possible to have reference data for some analyses. Whenever it was possible and pertinent, we used scales already published in the literature, for which the psychometric properties are already established.

#### Social and demographic characteristics

social position, educational and income level, employment and marital status, household composition, socioeconomic status of parents and spouse, and material living conditions (type of housing, household income, etc.), including geocoding of the residency address.

#### Health

personal and family history (cancer, cardiovascular, psychiatric); self-reported health scales (perceived health, quality of life, mental health, and specific scales for cardiovascular, musculoskeletal, and respiratory diseases); incident and prevalent diseases (from self-reports, social security long-term diseases and hospital discharge); information on sick leaves, handicaps, limitations, disabilities and injuries and healthcare utilization and management; and date and cause of death. In the HSC examination, weight, height, waist-hip ratio, blood pressure, electrocardiogram, vision, hearing, and lung function, laboratory tests (blood sugar level, lipid work-up, liver function tests, blood creatinine levels, complete blood counts, urine tests) will be measured.

#### Behavior

smoking and alcohol consumption (past and present), dietary habits and physical activity, marijuana use, sexual orientation.

#### Occupational factors

job history; lifelong and current occupational exposure to chemical, physical, and biological agents; postural, mechanical and organizational constraints; and stress at work.

#### Specific health problems of the elderly (45 years and older)

evaluation of functional capacities: IADL (Instrumental Activities of Daily Living) scale [36], questions from the French Handicaps-Disabilities-Impairments Survey [37], ability to use new technologies, and CASP (Control, Autonomy, Self-realisation and Pleasure [38], a quality of life scale particularly appropriate for senior citizens). Cognitive functions will be assessed through the MMSE [39], trail making A - B [40,41], Wechsler's coding subtest [42]; digital finger tapping [43], word fluency, formal lexical and semantic evocation [44,45], Grober & Busckhe's memory tests [46,47]; physical functioning through the gait speed [48], balance [49] and hand grip tests [50].

#### Biobank

we plan to collect biological samples (blood and urine) during visits to the HSC and to store the samples for each of the 200,000 participants. For blood, we will store 32 aliquots (0.5 ml) for each subject: 8 buffy-coat aliquots, 4 total blood aliquots (EDTA), 8 plasma aliquots on EDTA PST, and 8 plasma aliquots on Hep Li PST, 4 serum aliquots (Dry SST). For urine, we will keep 8 aliquots. In all, we plan to store about 8,000,000 aliquots. Standardized procedures for biological samples collection will be used, including standardized blood sampling (pre-treatment of the samples in each recruitment center within 30 mn after the collection), transport from each site to the central laboratory within the night (<24 h) at 4-8°C, robotised aliquoting in cryotubes (2D barcodes) in the central biorepository, and storage in deep freezers (-80°C).

In addition to this basic biobanking program, CONSTANCES will offer optional programs for specific research projects on subsets of participants, such as washed erythrocytes, RNA, proteins, mononuclear cells, saliva, or hair and nails.

### Periodicity of follow-up

The periodicity of follow-up will vary according to the sources. A self-administered mail questionnaire will be sent annually, thus allowing close follow-up, by collecting numerous data without asking subjects for too much work each year. At the same time, it will facilitate rapid response for setting up new studies and establish a sense of loyalty in the participants; too long a delay between two questionnaires is a factor that promotes dropping out [22]). Some data will be collected annually (health status and reported morbidity, life events and characteristics of place of residence, smoking, alcohol, etc.), while others will be collected at longer intervals, according to a planned calendar (health scales and questionnaires for a specific health area or specific risk factors). The mailing of self-administered questionnaires will be staggered over the year to take seasonal variations into account, since they are important for some topics (in particular, morbidity, drug use, and working conditions). Because the national databases essentially record events continuously, the follow-up of the data they provide will be permanent. Finally, participants will also be asked to come to the HSC every 5 years for medical and laboratory examinations.

### Quality control and validation of health events

The self-administered questionnaires will undergo the standard verifications: percentages of non-response, missing data, delay in return, etc.

For the data collected during the inclusion visit to the HSC, we established Standard Operational Procedures (SOP) for each of the examinations. Routine permanent quality control, based on regular on-site inspections by epidemiologic research assistants is planned, including monitoring of equipment used for the examinations, training of personnel, control of data completeness and validity from random samples of participants, etc. These quality control procedures will allow to assess the accuracy, reproducibility, concordance, and internal and external validity of the data collected and to study their factors of variability.

For the data extracted from the national databases, particular attention will be paid to validation of the diagnoses extracted from the health-related administrative databases, which will be routinely verified. Initially, we will particularly focus on some major outcomes: ischemic cardiovascular events, cancers, and neurodegenerative diseases. Each suspected outcome reported in the available sources will be routinely verified at the hospitals or with the general practitioners and validated by specialized expert committees.

## Results

### Pilot

A field pilot took place in seven of the participating HSCs from May 2009 to May 2010 for a four- to five-month period in each center. The pilot is not completely finished, and all data are not yet available. At the time this manuscript was prepared, about 3,500 subjects were included (women and men in almost equal numbers), and the preliminary analysis of the data showed that this sample was close to the general population of adults in France regarding sex, age and socioeconomic status. There was quite a diverse distribution of occupations and working conditions, lifestyle factors, and prevalence rates of various diseases and symptoms were close to those from other available French surveys. Regarding cognitive and physical functioning, there was also a very good variability in the tests results, in spite of the relatively young age of this sample.

Finally, the field pilot showed that the procedures for invitation of selected subjects, for data collection and medical examination were quite satisfactory, and that only some marginal adaptations of the protocol were necessary; these are currently being implemented. More detailed results of the field pilot can be found on the CONSTANCES website [35].

### Legal requirements

According to the French regulations, the CONSTANCES Cohort project has obtained the authorization of the National Data Protection Authority (*Commission nationale de l'informatique et des libertés*-CNIL). CNIL verified that before inclusion, clear information is provided to the eligible subjects (presentation of CONSTANCES, type of data to be collected, ability to refuse to participate, informed consent, etc.). Concrete procedures for setting up the two cohorts (participants and non-participants) ensure the confidentiality of the data at every point in its circulation as well as the anonymity of the cohort of non-participants. In addition, CONSTANCES was approved by the National Council for Statistical Information (*Conseil national de l'information statistique*-CNIS), the National Medical Council (*Conseil national de l'Ordre des médecins*-CNOM), and the Institutional Review Board of the National Institute for Medical Research-INSERM.

## Discussion

Considering its large size, the extensive coverage of the French adult population, the wealth of data collected from different sources, and its openness to the scientific community, CONSTANCES Cohort project should constitute a powerful tool for public health information and epidemiologic research in many different fields. The constitution of the full eligible sample is planned during the last trimester of 2010, and the inclusion of cohort participants will start at the beginning of 2011.

CONSTANCES has several strengths. It was designed both to provide public health information needed by the health authorities and to help answer research questions in diverse areas; to facilitate this goal, we devised a specific sampling scheme (including a non-participants cohort) and developed complex statistical procedures in order to take into account selection effects at inception as well as during the follow-up of the cohort. CONSTANCES will be a large cohort, including persons living and working in diverse settings, from large cities to small villages in different regions of France, with a broad range of socioeconomic status and trades. Numerous data will be collected at inception, including an extensive medical, physiological and biological examination, and a large biobank will be set up. The follow-up will be very extensive, relying both on active participation of the volunteers through annual questionnaires and regular visits to the HSCs, and on passive methods through the regular linkage to health and socioeconomic national exhaustive databases. Of particular importance is the high frequency of measurements from many different sources, allowing for analyses of lifecourse trajectories of health in relation to personal, social, occupational factors and major life events. Specific efforts were put into the quality of data collection and the validation of main outcomes in order to provide a highly phenotyped cohort. A unique feature of CONSTANCES is also to include a comprehensive set of cognitive and physical tests starting as young as 45 years, which is earlier in the lifecourse than most available studies on ageing.

The CONSTANCES has also some limitations. Due to the voluntary participation of cohort members, there will probably be an underrepresentation of hard-to-reach subjects, such as heavy drinkers or socially excluded persons. Comparisons between participants and non-participants at inclusion and during the follow-up through the "non-participants cohort" should allow assessment of potential biases due to selection effects, but lack of sufficient numbers in some categories might be a problem. Even more importantly, CONSTANCES will not offer sufficient power to study rare outcomes or exposures despite its large size. Simulations under several hypotheses regarding the prevalence of exposure and expected relative risk and duration of follow-up since inception, showed that in most of the situations where the relative risk is below 2, especially when interactions have to be taken into account, power will be satisfactory after at least 5 years of follow-up for situations where the incidence of the outcome is over 10/100,000 and the prevalence of exposure over 10% (data not shown). This limit is common to all longitudinal cohorts, and we are currently working with colleagues from other countries on a consortium for networking of prospective studies in Europe where several large-scale prospective cohorts already exist, or are currently under construction. The objective is to increase the standardization and collaboration between these various prospective study resources to meet goals of increasing the sample size and statistical power for multi-factorial risk analyses of infrequent outcomes.

## Competing interests

The authors declare that they have no competing interests.

## Authors' contributions

MZ and MG designed the full protocol and are the joint PIs of the CONSTANCES Cohort project; they also drafted the manuscript. AG, RS and JG designed the sampling protocol and elaborated the statistical procedures. MC, MN, AO, MCP, AQ and CR contributed to the design of the questionnaires, to the linkage with the national databases and the relationship with the HSCs. SB, GR and AS are in charge of the procedures for data collection, secured transmission and of the management of the CONSTANCES database. AB contributed to the development of the SOPs and the relationships with the participating HSCs. JH was in charge of the design of the biobank. All authors read and approved the final manuscript.

## Pre-publication history

The pre-publication history for this paper can be accessed here:

http://www.biomedcentral.com/1471-2458/10/479/prepub
